# The oldest record of Alvarezsauridae (Dinosauria: Theropoda) in the Northern Hemisphere

**DOI:** 10.1371/journal.pone.0186254

**Published:** 2017-10-25

**Authors:** Alexander Averianov, Hans-Dieter Sues

**Affiliations:** 1 Zoological Institute of the Russian Academy of Sciences, Universitetskaya nab. 1, Saint Petersburg, Russia; 2 Institute of Geology and Petroleum Technology, Kazan Federal University, Kazan, Russia; 3 Department of Paleobiology, National Museum of Natural History, Smithsonian Institution, Washington DC, United States of America; Institute of Botany, CHINA

## Abstract

Procoelous caudal vertebrae, a carpometacarpus with a hypertrophied metacarpal II, and robust proximal and ungual phalanges of manual digit II of a small theropod dinosaur from the Upper Cretaceous (Turonian) Bissekty Formation at Dzharakuduk, Uzbekistan, show unequivocal synapomorphies of the clade Alvarezsauridae and thus are referred to it. The caudal vertebrae have a unique longitudinal canal within the neural arch. The carpometacarpus, with metacarpal III occupying about one third of the width of the carpometacarpus, shows the most plesiomorphic stage of the evolution of the forelimb among known alvarezsaurids. The proximal phalanx of manual digit II differs from the corresponding bone in Parvicursorinae in having a less asymmetrical proximal articular surface without a dorsal process and short ventral ridges. The ungual phalanx of manual digit II has laterally open ventral foramina. The Bissekty alvarezsaurid possibly represents a basal parvicursorine and is the stratigraphically oldest known alvarezsaurid in Asia known to date.

## Introduction

Alvarezsauridae Bonaparte, 1991 is a clade of predominantly small-bodied, cursorial theropod dinosaurs with a suite of distinctive skeletal features, including opisthocoelous cervical and dorsal vertebrae, procoelous caudal vertebrae, short forelimbs with a hypertrophied olecranon process on the ulna, co-ossified carpometacarpus with a hypertrophied metacarpal II, robust manual digit II and reduced manual digits III-IV [1, 2]. This highly derived group comprises plesiomorphic North and South American taxa and a derived Asian clade, Parvicursorinae Karhu and Rautian, 1996 [3, 4]. Alvarezsaurids have also been reported from the uppermost Cretaceous (Maastrichtian) of Romania [5], but this attribution, based on a single incomplete tibiotarsus, is uncertain [4]. *Haplocheirus sollers* Choiniere et al., 2010 from the Upper Jurassic (Oxfordian) of Xinjiang (China), is considered a basal alvarezsauroid [6, 7]. It has unfused metacarpals and distal carpals and unreduced manual digits III-IV, lacking the specializations of Alvarezsauridae. This taxon is separated by an approximately 90 million-year gap from the Late Cretaceous alvarezsaurids in Asia, and the early stages of the evolutionary transformation of the highly modified manus of alvarezsaurids remain undocumented. The oldest known South American alvarezsaurid, the Turonian (~90 Mya) *Patagonykus puertai* Novas, 1994, already has a specialized forelimb with a modified humerus, forearm, and second manual digit [2, 8]. *Patagonykus puertai* differs from Parvicursorinae in the hypertrophied, hook-like proximoventral process of manual phalanx II-1. This possibly suggests a somewhat different use of the functionally monodactyl hand in *Patagonykus puertai* and the Asian Parvicursorinae. The stratigraphically oldest previously reported records of Alvarezsauridae in Asia are from the Coniacian-Santonian Majiacun Formation of Henan (China) (Table 1). An isolated fibula from the Coniacian-Santonian Iren Dabasu Formation of Inner Mongolia (China) has been referred to Alvarezsauridae [1] (see [9] concerning the stratigraphic age of the Iren Dabasu Formation). However, this bone more likely belongs to an avimimid oviraptorosaurian, which is common in these deposits [4]. Here we report the discovery of alvarezsaurid remains in the Turonian Bissekty Formation of Uzbekistan. The new specimens are stratigraphically oldest known records of this clade in Asia and the Northern Hemisphere. They include carpometacarpi that document a previously unknown stage in the forelimb evolution among Alvarezsauridae.

**Table 1:** Spatial and temporal distribution of Alvarezsauridae.

Taxon	Country	Formation	Age	References
*Mononykus olecranus* Perle et al., 1991	Mongolia	Nemegt Formation (Bugin Tsav)	Maastrichtian	[21]
Alvarezsauridae indet.	Montana, USA	Hell Creek Formation	Maastrichtian	[28]
Alvarezsauridae indet.	Wyoming, USA	Lance Formation	Maastrichtian	[29, 30]
*Albertonykus borealis* Longrich and Currie, 2009	Alberta, Canada	Horseshoe Canyon Formation	Maastrichtian	[4]
*Bonapartenykus ultimus* Agnolin et al., 2012; Alvarezsauridae indet.	Argentina	Allen Formation	Campanian-Maastrichtian	[26, 31]
*Parvicursor remotus* Karhu and Rautian, 1996	Mongolia	Baruungoyot Formation (Khulsan)	Campanian	[25]
*Ceratonykus oculatus* Alifanov and Barsbold, 2009	Mongolia	Baruungoyot Formation (Khermiin Tsav)	Campanian	[32]
*Shuvuuia deserti* Chiappe et al., 1998*, *Kol ghuva* Turner et al., 2009**	Mongolia	Djadokhta Formation (Ukhaa Tolgod, Tugrikin-Shire)	Campanian	[22, 33, 34]
*Linhenykus monodactylus* Xu et al., 2011; *Linhenykus* sp.; Alvarezsauridae indet.	Inner Mongolia, China	Wulansuhai Formation (Bayan Mandahu)	Campanian	[3, 14, 35, 36]
*Albinykus baatar* Nesbitt et al., 2011	Mongolia	Javkhlant Formation	?Santonian—Campanian	[37]
*Alvarezsaurus calvoi* Bonaparte, 1991; *Achillesaurus manazzonei* Martinelli and Vera, 2007	Argentina	Bajo de la Carpa Formation	Santonian	[20, 23]
*Xixianykus zhangi* Xu et al., 2010	Henan, China	Majiacun Formation	Coniacian-Santonian	[27]
*Patagonykus puertai* Novas, 1994	Argentina	Portezuelo Formation	Turonian	[2, 8, 24]
Alvarezsauridae indet.	Uzbekistan	Bissekty Formation	Turonian	This report
*Alnashetri cerropoliciensis* Makovicky et al., 2012	Argentina	Candeleros Formation	Cenomanian-Turonian	[38]

* The specimen from Tugrikin-Shire possibly represents a distinct taxon similar to *Parvicursor* [4].

** Based on a single articulated pes that may belong to an oviraptorosaurian [26].

## Methods

**Fig 1:**
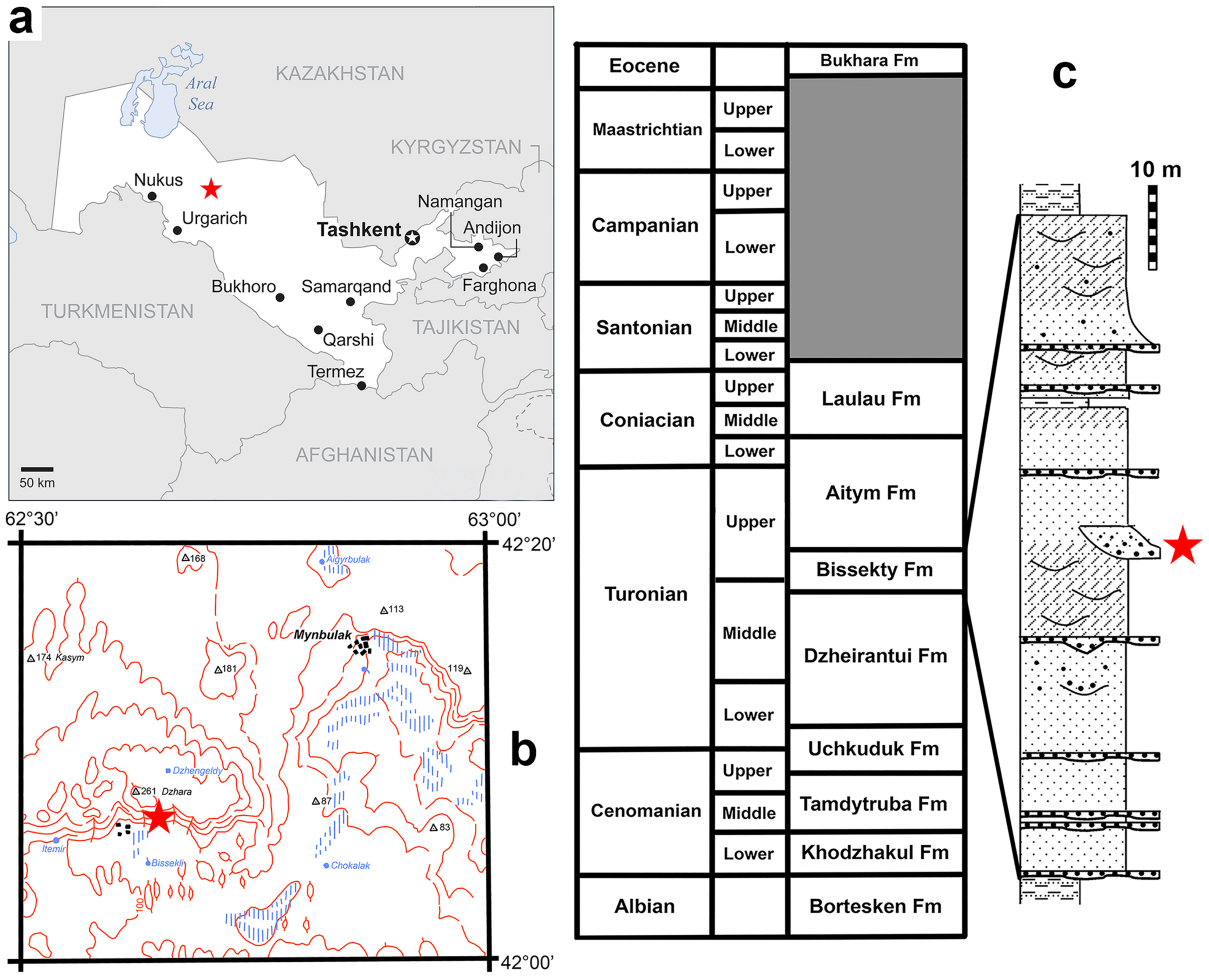
Location of the Dzharakuduk locality complex (indicated by star) on a map of Uzbekistan and neighboring regions (**a**) and on a more detailed map of the region around Mynbulak (**b**). Stratigraphic scheme of the Cretaceous strata in the central Kyzylkum Desert, Uzbekistan with a section of the Bissekty Formation at Dzharakuduk, with the position of the site CBI-14 marked by an asterisk (**c**).

The specimens described here were collected by Lev A. Nesov and colleagues in 1977–1994 and by the URBAC (Uzbekistan/Russian/British/American/Canadian) joint paleontological expeditions in 1997–2006 in the Turonian Bissekty Formation at Dzharakuduk, central Kyzylkum Desert, Uzbekistan [10, 11]. At Dzharakuduk, the Bissekty Formation is exposed along an escarpment that extends from about 42°06'22.60'' N and 62°37'09.00'' E to 42°05'44.22'' N and 62°41'06.49'' E (Fig 1b). The Bissekty Formation (Fig 1c) comprises an up to 80 m thick succession of medium-grained, poorly lithified, cross-bedded fluvial sandstones and clast-supported, well-cemented intraformational conglomerates [12]. The geological age is bracketed using invertebrate fossils from marine units overlying and underlying the Bissekty Formation as well as based on comparisons with the Late Cretaceous vertebrate assemblages of Central Asia [9]. The unit is assigned a middle to late Turonian age, approximately ~93–90 Ma [13]. Fossils were recovered by surface collecting at the most fossiliferous sites in 1977–1994 with subsequent dry and wet screening of 300 metric tons of matrix between 1997 and 2006.

Alvarezsaurid remains are the rarest dinosaurian fossils in the Dzharakuduk faunal assemblage. There are only seven alvarezsaurid bones known out of some 3500 cataloged dinosaurian specimens from Dzharakuduk. Alvarezsaurid bones were collected from sites CBI-14 and CBI-4 where the most intensive screen-washing of fossil-bearing sediments was undertaken. This highlights the importance of screen-washing large quantities of matrix for the recovery of rare faunal elements.

The locality information following catalog numbers uses prefixes established by Nesov for localities within the middle-upper of the Bissekty Formation: CBI—Central (Kyzylkum) Bissekty.

Carpal terminology follows [14]. The digits in the tetanuran manus are interpreted as II-IV following [15].

## Systematic paleontology

Dinosauria Owen, 1842 [16]

Saurischia Seeley, 1887 [17]

Theropoda Marsh, 1881 [18]

Maniraptora Gauthier, 1986 [19]

Alvarezsauridae Bonaparte, 1991 [20]

Alvarezsauridae gen. et sp. indet.

### Material

ZIN PH 2441/16 (CBI-4e), two articulated posterior caudal vertebrae; ZIN PH 2442/16 (CBI-14), posterior caudal vertebra missing the ventral portion of the centrum; ZIN PH 2440/16 (CBI-14), right carpometacarpus; ZIN PH 2443/16 (CBI-4), left carpometacarpus; ZIN PH 2444/16 (CBI-14), left manual phalanx II-1; ZIN PH 2445/16 (CBI-), ungual phalanx of right manual digit II; ZIN PH 2446/16 (CBI-4), poorly preserved ungual phalanx of manual digit II (side uncertain).

### Locality and horizon

Dzharakuduk, central Kyzylkum Desert, Uzbekistan; Bissekty Formation; Upper Cretaceous (middle-upper Turonian).

### Description

#### Caudal vertebrae

**Fig 2:**
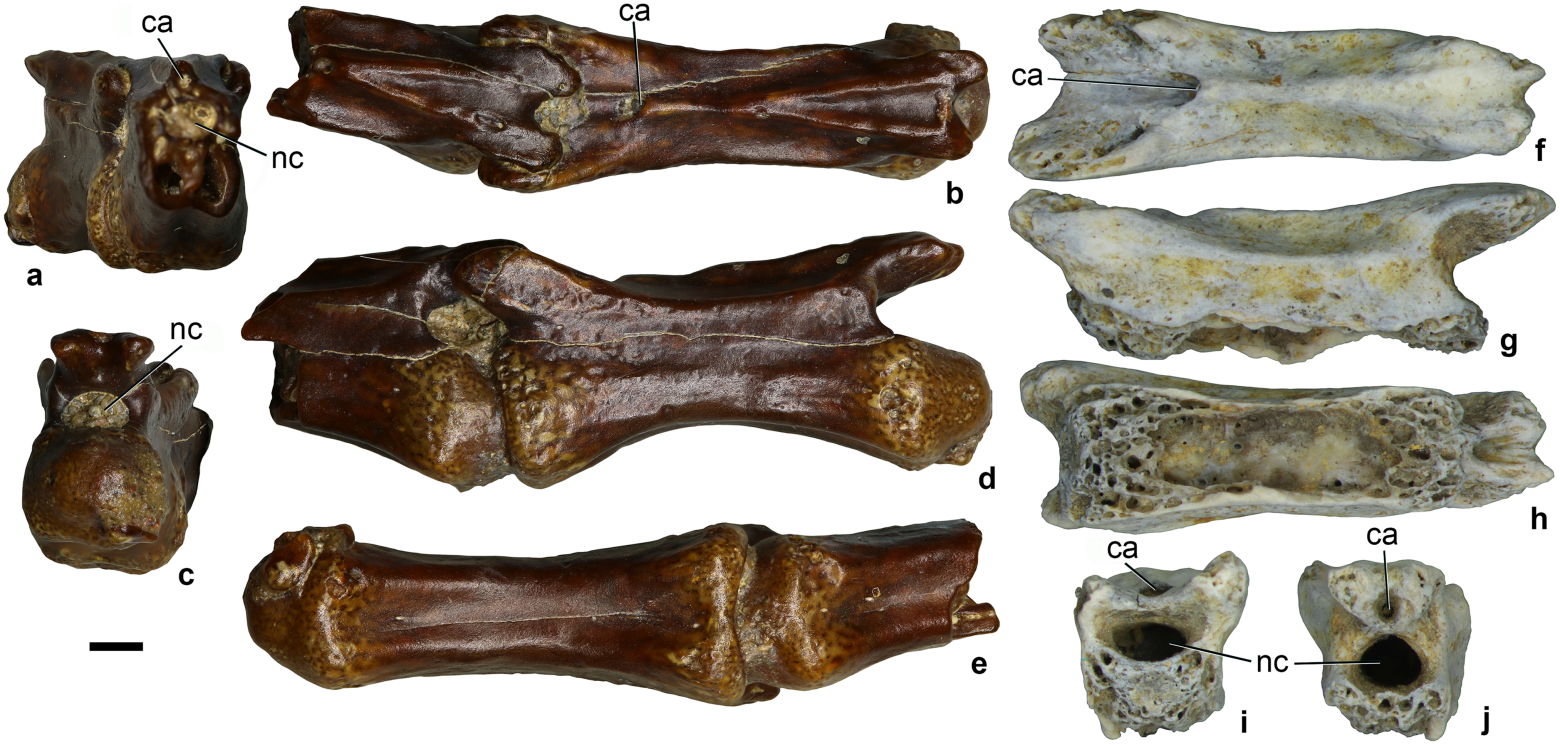
Alvarezsauridae gen. et sp. indet., posterior caudal vertebrae. Dzharakuduk, Uzbekistan; Bissekty Formation, Upper Cretaceous (Turonian). **a-e**, ZIN PH 2441/16, two vertebrae preserved in articulation, in anterior (**a**), dorsal (**b**), posterior (**c**), lateral (**d**), and ventral (**e**) views. **f-j**, ZIN PH 2442/16, vertebra missing ventral part of the centrum, in dorsal (**f**), lateral (**g**), ventral (**h**), anterior (**i**), and posterior (**j**).

The posterior caudal vertebrae are procoelous and elongate, with a ratio of centrum length to anterior centrum width of 2.91 (Fig 2). The cotyle and condyle of the centrum are round in outline in end view. The condyle is hemispherical. In lateral view, the centrum has a deeply concave ventral margin. Its ventral surface bears a shallow longitudinal groove, which is flanked by low ridges laterally. The centrum is almost quadrangular in cross-section and hollow. The neural canal is oval and dorsoventrally compressed in outline. The neural arch lacks a neural spine and transverse processes. The prezygapophyses project anteriorly beyond the centrum whereas the postzygapophyses are in line with the posterior end of the centrum. The articular surfaces of the pre- and postzygapophyses are oval in outline. Faint ridges border a broad prespinal depression and connect the prezygapophyses to a point corresponding to the position of a neural spine on other vertebrae. Anterior to that point, a small foramen is connected to the longitudinal groove within the prespinal depression. This foramen opens into a longitudinal canal within the neural arch. This canal opens posteriorly between the closely spaced postzygapophyses in ZIN PH 2442/16 (Fig 2j) but is closed posteriorly in ZIN PH 2441/16 (Fig 2c). In the latter specimen, the canal is visible in mid-section on the broken vertebra (Fig 2a). At the posterior part of the neural arch, there is a triangular depression bordered by distinct ridges. About half of this depression is above the postzygapophyses. A distinct median ridge extends between the prespinal depression and the triangular depression. A pronounced ridge is developed between the pre- and postzygapophysis.

#### Carpometacarpus

**Fig 3:**
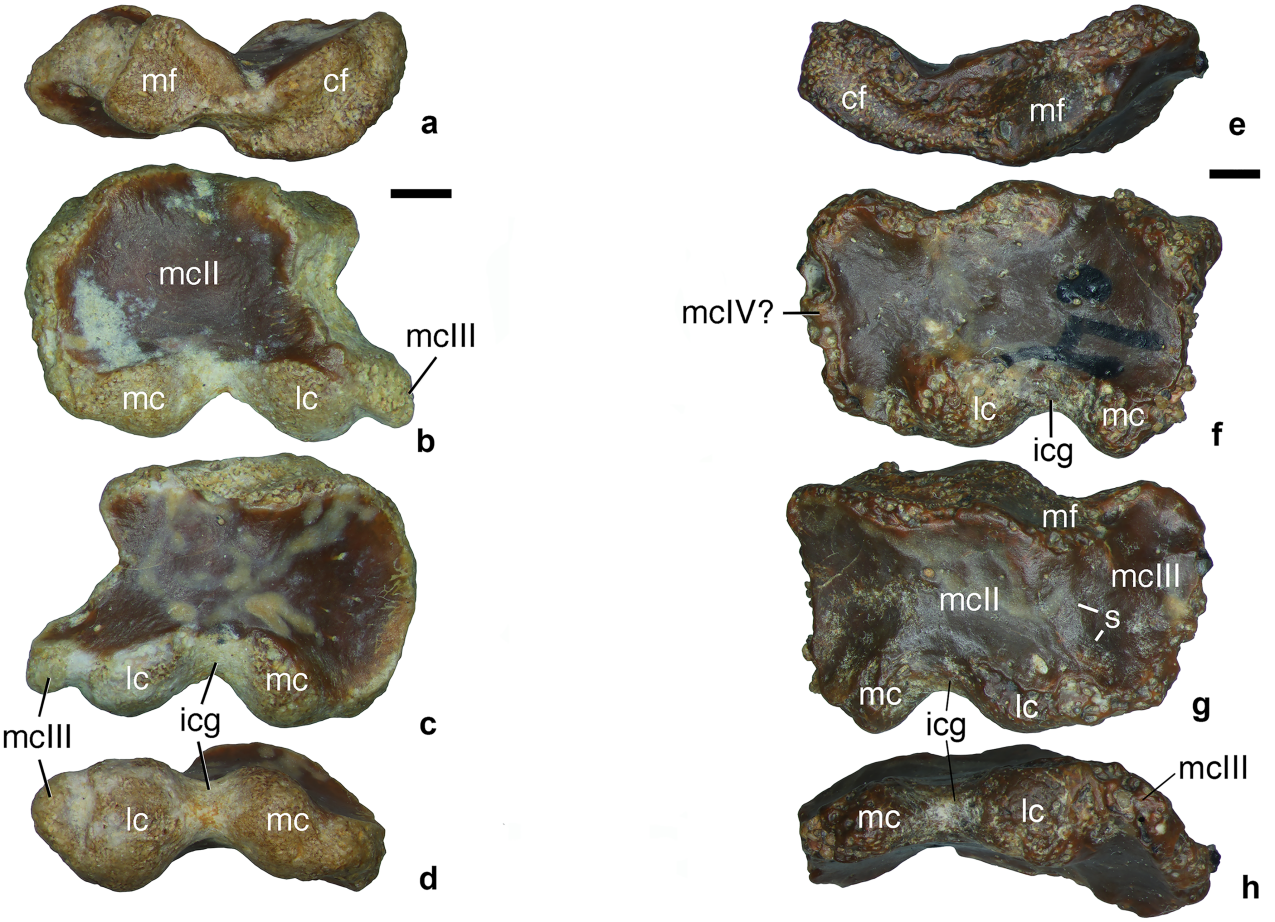
Alvarezsauridae gen. et sp. indet., carpometacarpus. Dzharakuduk, Uzbekistan; Bissekty Formation, Upper Cretaceous (Turonian). **a-d**, ZIN PH 2440/16, right carpometacarpus, in proximal (**a**), ventral (**b**), dorsal (**c**), and distal (**d**) views. **e-h**, ZIN PH 2443/16, left carpometacarpus, in proximal (**e**), ventral (**f**), dorsal (**g**), and distal (**h**) views. Abbreviations: cf, central facet; icg, intercondylar groove; lc, lateral condyle; mc, medial condyle; mf, medial facet; mcII, metacarpal II; mcIII, metacarpal III; mcIV?, possible metacarpal IV; s, suture between metacarpals II and III. Scale bars equal 1 mm.

Two carpometacarpi have been recovered. ZIN PH 2440/16 represents a right carpometacarpus consisting of the complete metacarpal II and possibly incorporating the semilunar carpal [21] and the distal end of metacarpal III indistinguishably fused with metacarpal II (Fig 3a–3d). ZIN PH 2443/16 is a left carpometacarpus, about 33% larger than ZIN PH 2440/16, and its metacarpal III is completely fused with metacarpal II (Fig 3e–3h). A tiny metacarpal IV may have also been present but details are not clear. In ZIN PH 2440/16, only the distal part of metacarpal III was fused to metacarpal II whereas the more proximal portion of metacarpal III was not, with a contact surface on metacarpal II. The flattened metacarpal II is convex dorsally and concave ventrally. On the dorsal surface, the two planes meet along the midline but there is no distinct longitudinal ridge as in *Patagonykus puertai* [8]. In dorsal/ventral view, metacarpal II has a subtrapezoidal outline with convex medial and proximal margins and a concave lateral margin. On its proximal margin, there two distinct articular surfaces, the central and the medial facets. The medial facet is triangular and convex. In ZIN PH 2443/16, the medial facet is clearly divided into two planes, which are set at an angle and correspond to the contacts for metacarpals II and III. In ZIN PH 2440/16, only part of this facet, corresponding to the contact with metacarpal III, is present. The central facet is bean-shaped and also convex. The distal margin of the bone is occupied by the ginglymoid articular surface for the proximal phalanx of manual digit II. The distal articular surface is clearly defined with sharp margins and almost equally exposed on the dorsal and ventral surfaces of the bone. The lateral condyle of metacarpal II is globular, with its main axis extending perpendicular to the dorsoventral plane. The medial condyle is more compressed dorsoventrally and has an obliquely aligned main axis. The angle between the axes of the two condyles is about 63° (ZIN PH 2440/16). A wide intercondylar groove separates the two condyles. Both distal condyles of metacarpal II project almost equally distally. In ZIN PH 2440/16, metacarpal III has the distal articular condyle separated from the lateral condyle of metacarpal II by a groove. This groove is wide on the dorsal surface of the bone and tapers towards the ventral surface. The articular condyle of metacarpal III is eroded on ZIN PH 2443/16. On that specimen, metacarpal III constitutes about one third of the width of the carpometacarpus, much more than in *Mononykus olecranus* [21]. There is a faint trace of the separation between metacarpals II and III on the dorsal side of ZIN PH 2443/16.

#### Manual phalanx II-1

**Fig 4:**
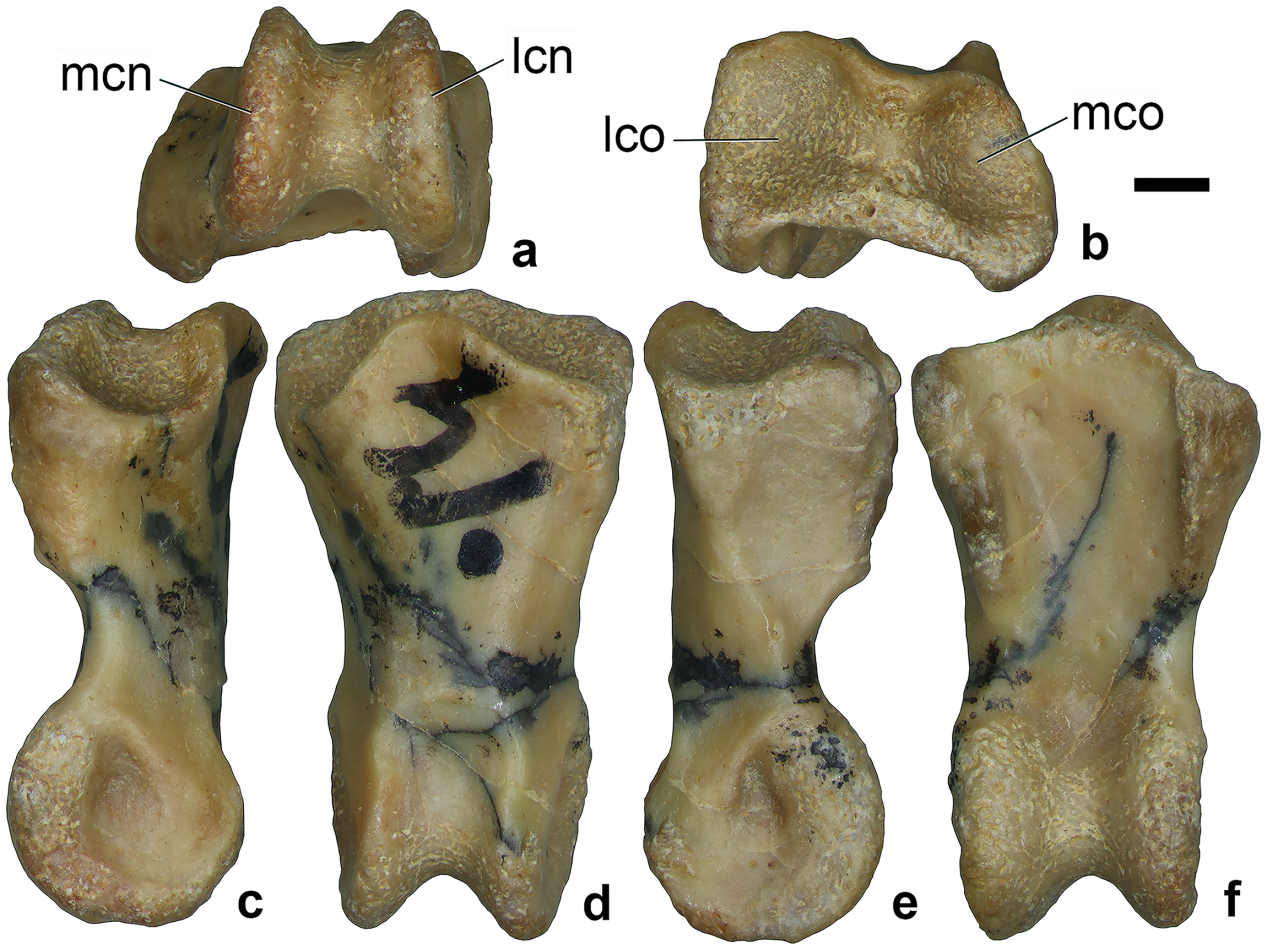
Alvarezsauridae gen. et sp. indet., ZIN PH 2444/16, left manual phalanx II-1, in distal (a), proximal (b), medial (c), dorsal (d), lateral (e), and ventral (f) views. Dzharakuduk, Uzbekistan; Bissekty Formation, Upper Cretaceous (Turonian). Abbreviations: lcn, lateral condyle; lco, lateral cotyle; mco, medial cotyle; mcn, medial condyle. Scale bar equals 1 mm.

The left proximal phalanx of manual digit II (ZIN PH 2444/16; Fig 4) perfectly articulates with the carpometacarpus ZIN PH 2443/16. Both specimens appear to represent fully grown individuals. The proximal articular surface of the phalanx is asymmetrical (Fig 4b), with its proximal cotyles separated by a ridge and mirroring the shape of the distal condyles of metacarpal II. The lateral cotyle is more globular whereas the medial cotyle is more oblique. The dorsal side bears a distinct subcircular depression between the distal condyles (Fig 4d), as in *Mononykus olecranus* Perle et al., 1991 [21] and *Patagonykus puertai* [8]. On the ventral side, there are lateral ridges connected with the proximal articular surfaces (Fig 4f). These ridges extend for about half the length of the phalanx. The medial ridge is the more prominent of the two. On the side, a distinct oblique ridge connects the proximal articular surfaces to the lateral distal condyle (Fig 4e). On the distal end of the phalanx, a narrow intercondylar groove separates the well-developed condyles of the ginglymus. The lateral condyle is slightly larger and extends more distally than the medial one. There are distinct, teardrop-shaped pits for the collateral ligaments on the lateral and medial aspects of the distal end of the phalanx. The lateral pit is slightly deeper than the medial one.

#### Manual phalanx II-2 (ungual)

**Fig 5:**
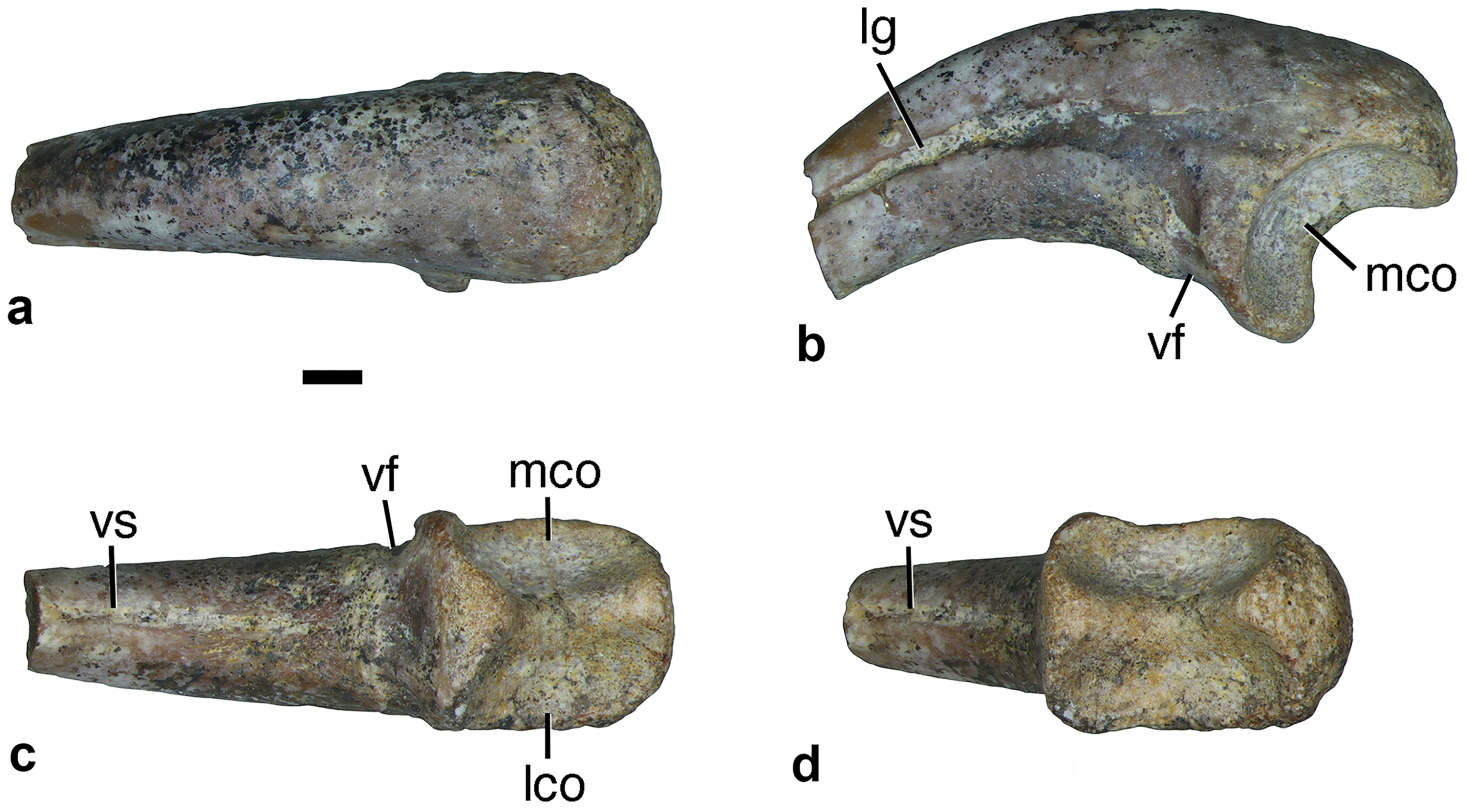
Alvarezsauridae gen. et sp. indet., ZIN PH 2445/16, right manual phalanx II-2 (ungual), in dorsal (a), lateral (b), ventral (c), and proximal (d) views. Dzharakuduk, Uzbekistan; Bissekty Formation, Upper Cretaceous (Turonian). Abbreviations: lco, lateral cotyle; lg, lateral groove; mco, medial cotyle; vf, ventral foramen; vs, ventral sulcus. Scale bar equals 1 mm.

ZIN PH 2445/16 is a nearly complete, well-preserved ungual phalanx of a right manual digit II (Fig 5). It closely matches the size of the preungual phalanx ZIN PH 2444/16. ZIN PH 2446/16 is a poorly preserved ungual of a manual digit II, which does not differ in observable details from ZIN PH 2445/16. The ungual is strongly curved. If its long axis is aligned horizontally, the proximal articular surface faces proximoventrally (Fig 5b). The proximal articular surface is slightly asymmetrical, with the lateral cotyle being slightly larger than the medial one. A robust ridge separates the cotyles. The ungual lacks a flexor tubercle or a dorsal lip. The lateral groove becomes deeper distally. The ventral rim of the groove does not protrude above the level of the dorsal rim. More proximally, the lateral groove divides into two branches. One branch continues proximally whereas the other branch extends to the ventral surface of the phalanx where it forms a distinct lateral groove (‘ventral foramen’ in Fig 5b and 5c). A distinct but shallow ventral sulcus is present along the midline on the ventral surface of the ungual (Fig 5c and 5d).

### Measurements

Caudal vertebra ZIN PH 2440/16: anterior height of centrum (ACH) 2.7 mm; anterior width of centrum (ACW) 3.4 mm; anterior width of neural arch (between lateral margins of prezygapophyses) (ANW) 5.6 mm; centrum length (CL) 9.9 mm; posterior height of centrum (PCH) 2.6 mm; posterior centrum width (PCW) 3.0 mm; posterior width of neural arch (between lateral margins of postzygapophyses) (PNW) 2.2 mm.

Carpometacarpus ZIN PH 2440/16: maximum proximodistal length 4.3 mm; maximum mediolateral width (metacarpal II + III) 6.4 mm. ZIN PH 2443/16: maximum proximodistal length 5.7 mm; maximum mediolateral width (metacarpal II + III) 8.4 mm.

Manual phalanx II-1 (ZIN PH 2444/16): length 8.6 mm; proximal width 4.8 mm; distal width 3.5 mm.

Manual phalanx II-2 (ungual; ZIN PH 2445/16): proximal width 3.7 mm.

### Comparisons

Posterior caudal vertebrae of Alvarezsauridae are best known in *Shuvuuia deserti* Chiappe et al., 1998 [22]. These vertebrae closely resemble those from the Bissekty Formation in having elongate centra and short prezygapophyses, in the absence of a neural spine, the presence of a sulcus extending along the entire length of the ventral surface of the centrum, and the presence of a triangular depression on the posterior part of the neural arch. The longitudinal canal within the neural arch has not previously been described for alvarezsaurid caudals, but it appears to be present in *Shuvuuia deserti* based on a photograph (fig. 4 in [22]). This canal was also figured on the anterior caudals of *Achillesaurus manazzonei* Martinelli and Vera, 2007 [23] but incorrectly labeled “neural canal” (fig. 3B in [23]); the actual neural canal extends between the centrum and neural arch. The posterior caudal vertebrae of *Alvarezsaurus calvoi* Bonaparte, 1991 (fig. 25C in [20]) resemble those of the Bissekty alvarezsaurid in their strongly convex ventral profile.

Among the alvarezsaurids, the carpometacarpus has been described for *Patagonykus puertai*, *Mononykus olecranus*, and *Linhenykus monodactylus* Xu et al., 2011 [1, 2, 8, 14, 21]. In ZIN PH 2443/16, metacarpal III is much less reduced than in the latter two taxa. In *Patagonykus puertai*, the medial margin of metacarpal II is concave, whereas in *Mononykus olecranus*, *Linhenykus monodactylus*, and the Bissekty carpometacarpi, it is strongly convex (ZIN PH 2440/16) or straight (ZIN PH 2443/16). The dorsal surface of metacarpal II of *Patagonykus puertai* bears a longitudinal ridge, which is absent in *Mononykus olecranus* and weakly developed on the Bissekty carpometacarpi. In *Patagonykus puertai* and *Linhenykus monodactylus*, the distal articular surface of metacarpal II is more extensively exposed on the dorsal surface of the bone than on the ventral one. In *Mononykus olecranus* and the Bissekty carpometacarpi, the dorsal and ventral articular surfaces of the distal trochlea are nearly equally exposed. In *Patagonykus puertai* and *Linhenykus monodactylus*, the lateral condyle of the distal trochlea projects more distally than the medial condyle, whereas both condyles equally project distally in *Mononykus olecranus* and the Bissekty carpometacarpi. In *Patagonykus puertai*, unlike in the Bissekty carpometacarpi, the lateral condyle of the distal trochlea of metacarpal II is not globular and its long axis does not extend perpendicular to the dorsoventral plane. In *Mononykus olecranus*, both condyles of the distal trochlea of metacarpal II are more compressed dorsoventrally than in the Bissekty carpometacarpi. Metacarpal II of *Linhenykus monodactylus* is much narrower transversely than those of *Mononykus olecranus* and the Bissekty carpometacarpi. The Bissekty carpometacarpi lack the accessory medial facet present in *Linhenykus monodactylus* and *Mononykus olecranus* [14]. The carpometacarpus is also known, but has not yet been described in detail, for *Shuvuuia deserti*. In the latter, metacarpals II and III are not co-ossified [1].

The proximal phalanx of manual digit II of the Bissekty alvarezsaurid is proportionally longer and less asymmetrical than that in *Mononykus olecranus* and lacks the dorsal process on the proximal side. ZIN PH 2444/16 is similar to manual phalanx II-1 in the latter taxon in the moderate development of the ventral ridges, whereas the lateral ridge is hypertrophied in the hook-like proximoventral process in *Patagonykus puertai* [1, 2, 8, 24]. In ZIN PH 2444/16, the distal condyles are more asymmetrical compared with those in *Patagonykus puertai*, but less asymmetrical than in *Mononykus olecranus*. In the latter taxon, the ventral ridges of the manual phalanx II-1 extend further distally than in ZIN PH 2444/16.

The ungual phalanx of manual digit II (phalanx II-2) is almost identical in its proportions to that in *Albertonykus borealis* Longrich and Currie, 2009 (fig. 4 in [4]). Both bones are also similar in the presence of a proximal division of the lateral groove to form a shallow “Y”. This feature has been reported as absent in the Asian and South American alvarezsaurids [4]. However, at least in *Mononykus olecranus*, this character might be obscured by a bony bridge above the lateral groove that forms the lateral wall of the ventral foramen (fig. 14B in [21]). The lateral groove is not bifurcated proximally in *Linhenykus monodactylus*, where the bony bridge is absent and the ventral foramen opens laterally (fig. 9F in [14]), as in the Bissekty alvarezsaurid. The lateral groove is not bifurcated proximally and the ventral foramina are open ventrally (“ventroproximal notch”) on the manual ungual of *Patagonykus puertai* (fig. 2 in [24]). The manual unguals of *Alvarezsaurus calvoi* (fig. 4A-C in [2]) and *Patagonykus puertai* (fig. 2B in [24]) differ from those in other alvarezsaurids, including the Bissekty form, in the presence of a ventral ridge (flexor tubercle). The lateral groove can be bifurcated proximally on pedal unguals in some alvarezsaurids (Fig. 31F in [8]).

In ZIN PH 2445/16, the proximal articular surface is displaced to the ventral side of the ungual whereas it occupies a terminal position in *Mononykus olecranus* (fig. 14 in [21]). It also differs from the latter in the less developed flattened area on the ventral surface around the ventral foramina. The ventral sulcus in ZIN PH 2445/16 is also present in *Mononykus olecranus*, *Albertonykus borealis*, and *Linhenykus monodactylus* [4, 14, 21], and might prove to be a shared feature for all alvarezsaurids.

## Discussion

The skeletal specializations of Alvarezsauridae are so distinctive that this taxon can be readily identified based even on a few isolated bones. The material from the Bissekty Formation referred to Alvarezsauridae shows the following synapomorphies for this clade: procoelous caudal vertebrae; hypertrophied and strongly depressed metacarpal II; and robust manual digit II with ungual phalanx bearing two proximodistal foramina [1]. The diagnostic characters of the Asian alvarezsaurid subclade, Parvicursorinae Karhu and Rautian, 1996, mostly concern the pelvic girdle and hind limb [1, 3, 14, 25–27] and thus cannot be assessed for the available alvarezsaurid remains from the Bissekty Formation. Parvicursorines were more adapted for a cursorial mode of life than the South American alvarezsaurids, as exemplified by the “arctometatarsalian” structure of the pes and the distally reduced fibula.

The Asian alvarezsaurids differ from *Patagonykus puertai* by the strongly asymmetrical proximal articular surface of manual phalanx II-1, with its dorsolateral corner projecting dorsally [1] (dorsal process [21]). The Bissekty alvarezsaurid is more similar in this respect to *Patagonykus puertai*: the proximal articular surface of manual phalanx II-1 is less asymmetrical and lacks the dorsal process. This is apparently a plesiomorphic condition for Alvarezsauridae. The Bissekty alvarezsaurid differs from *Patagonykus puertai* in the absence of hypertrophied, hook-like proximoventral process of manual phalanx II-1. Thus, the Bissekty alvarezsaurid is probably not referable to Patagonykinae Agnolin et al., 2012 [26].

**Fig 6:**
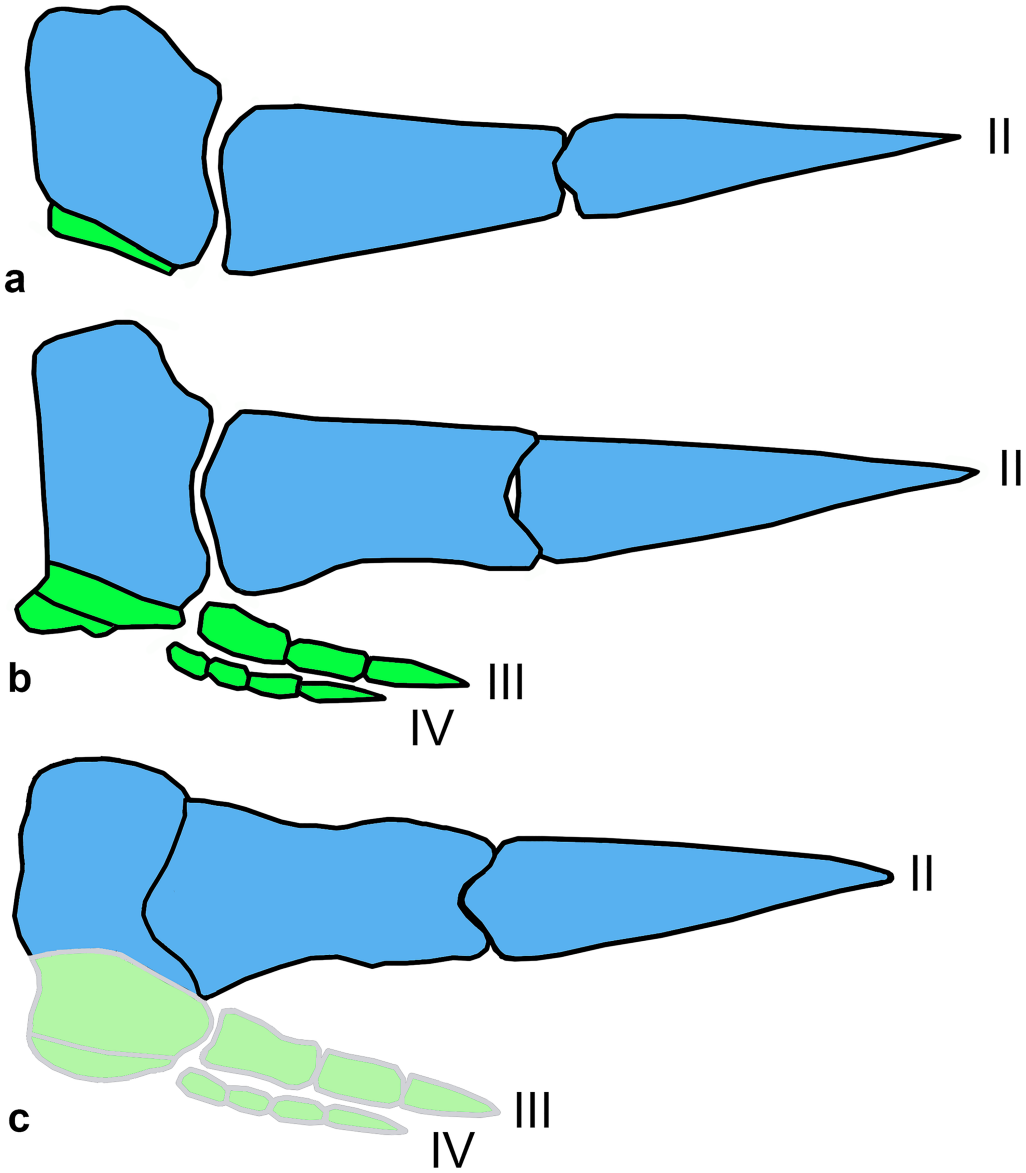
Diagrams illustrating stages in the reduction of the manus among Alvarezsauridae (right manus in dorsal view; II-IV—manual digits II-IV; manual digit II is shown in blue and manual digits III and IV in green). **a**, *Linhenykus monodactylus*; **b**, *Shuvuuia deserti* and *Mononykus olecranus*; **c**, Bissekty alvarezsaurid (reconstructed manual digits III and IV indicated in lighter shade). **a** and **b** redrawn from [3].

The previously oldest known Asian alvarezsaurid, the Coniacian-Santonian *Xixianykus zhangi* Xu et al., 2010, combined the plesiomorphic structure of the synsacrum and pelvis with a fully “arctometatarsalian” pes [27]. The Campanian *Linhenykus monodactylus*, with the most derived known structure of the forelimb among alvarezsaurids (Fig 6), was surprisingly placed as the basalmost parvicursorine in some phylogenetic analyses [3, 14]. The Turonian Bissekty alvarezsaurid cannot be unequivocally attributed to Parvicursorinae because the structure of its pelvis and hind limb remains unknown. However, attribution to this group is plausible because only parvicursorines are known from the Late Cretaceous of Asia to date. The Bissekty form is possibly one of the most basal parvicursorines based on its relatively unmodified metacarpal III, which occupies about one third of the width of the carpometacarpus (Fig 6), the less asymmetrical proximal articular surface of manual phalanx II-1 and the absence of a dorsal process, the presence of short ventral ridges on that phalanx, and the laterally open ventral foramina on the ungual of manual digit II. The Bissekty alvarezsaurid has a distinctive canal within the neural arch of the posterior caudal vertebrae, but this feature may have had a wider distribution among Alvarezsauridae.

The current paleobiogeographic scenario for Alvarezsauridae postulates that the clade Alvarezsauria originated in Laurasia (Asia) during the Late Jurassic. During the Late Cretaceous, Alvarezsauridae originated in Gondwana (South America) and dispersed to Laurasia where Parvicursorinae differentiated [2–4, 6, 8, 28]. Some authors have hypothesized that alvarezsaurids initially migrated from Gondwana to North America and then to Asia [2, 4]. Other researchers suggested that derived Parvicursorinae came to North America from Asia, where this group was abundant during the Campanian and Maastrichtian [3, 14, 26, 27]. The discovery of an alvarezsaurid in the Turonian of Uzbekistan indicates that this group had a longer evolutionary history in Asia and the origin of this clade might even be connected to this continent.
